# Nanoscale potassium niobate crystal structure and phase transition

**DOI:** 10.1186/1556-276X-6-530

**Published:** 2011-09-23

**Authors:** Haiyan Chen, Yixuan Zhang, Yanling Lu

**Affiliations:** 1 Institute of Marine Materials Science and Engineering, Shanghai Maritime University, 1550 Harbor Avenue, Lingang New City, Shanghai 201306, China; 2 State Key Laboratory of Metal Matrix Composites, Shanghai Jiaotong University, 800 Dongchuan Road, Shanghai 200240, China; 3 Shanghai Institute of Applied Physics, Chinese Academy of Sciences, 239 Zhangheng Road, Shanghai 201204, China

**Keywords:** potassium niobate, crystal structure, phase transition, nanoscale powder.

## Abstract

Nanoscale potassium niobate (KNbO_3_) powders of orthorhombic structure were synthesized using the sol-gel method. The heat-treatment temperature of the gels had a pronounced effect on KNbO_3 _particle size and morphology. Field emission scanning electron microscopy and transmission electron microscopy were used to determine particle size and morphology. The average KNbO_3 _grain size was estimated to be less than 100 nm, and transmission electron microscopy images indicated that KNbO_3 _particles had a brick-like morphology. Synchrotron X-ray diffraction was used to identify the room-temperature structures using Rietveld refinement. The ferroelectric orthorhombic phase was retained even for particles smaller than 50 nm. The orthorhombic to tetragonal and tetragonal to cubic phase transitions of nanocrystalline KNbO_3 _were investigated using temperature-dependent powder X-ray diffraction. Differential scanning calorimetry was used to examine the temperature dependence of KNbO_3 _phase transition. The Curie temperature and phase transition were independent of particle size, and Rietveld analyses showed increasing distortions with decreasing particle size.

## Background

Lead oxide-based perovskites are a commonly used piezoelectric material and are now widely used in transducers and other electromechanical devices [1-4]. However, the high toxicity and high processing vapor pressure of lead oxide cause serious environmental problems. A promising way to address this issue is to develop lead-free piezoelectric ceramics to minimize lead pollution. Recently, as demand has increased, many studies have focused on the development of high-quality lead-free piezoelectric materials [5-7].

Potassium niobate (KNbO_3_) is a ferroelectric compound with a perovskite-type structure and is a promising piezoelectric material owing to superior coupling in its single crystal form [8,9]. KNbO_3 _materials have attracted considerable attention for applications in lead-free piezoelectric materials. KNbO_3 _has an orthorhombic structure and is a well-known ferroelectric material with extensive applications in electromechanical, nonlinear optical, and other technological fields [10-13].

KNbO_3 _phase transition temperatures have already been determined. KNbO_3 _can exist in orthorhombic, tetragonal, and cubic phases above room temperature, and at ambient pressure, it exhibits two structural transitions with decreasing temperature: cubic to tetragonal at 691 K and tetragonal to orthorhombic at 498 K [14]. The cubic phase is paraelectric while the remaining two are ferroelectric; however, phase transitions of nanoscale KNbO_3 _have not yet been reported in detail.

The phase transition temperatures of ferroelectric ceramics are size dependent, with the ferroelectric phase becoming unstable at room temperature when the particle diameter decreases below a critical size [15-17]. However, this critical size usually encompasses a broad size range. Experimental discrepancies may arise because of intrinsic differences between ferroelectric samples, and several theoretical models based on Landau theory overestimate the critical sizes [18]. Therefore, the phase structure of nanoscale KNbO_3 _at room temperature requires further investigations.

The current work is a systematic study of the crystal structure and phase transitions of nanoscale KNbO_3_, synthesized using the sol-gel method. The aim was to investigate the size dependence of the ferroelectric phase and the phase transition temperatures of nanoscale KNbO_3 _powders.

## Results and discussion

**Figure 1:**
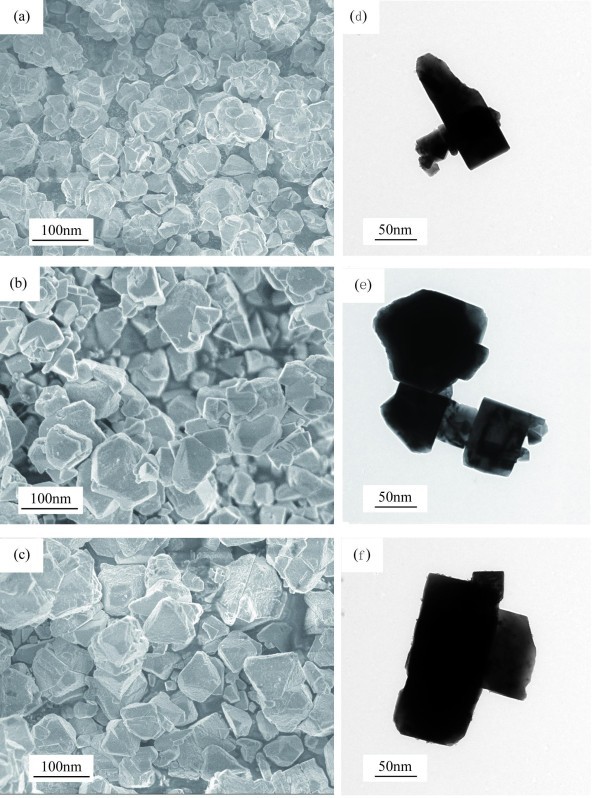
**FESEM images of nanoscale KNO_3 _powders obtained by heat-treating gels**. At (**a**) 600°C, (**b**) 700°C, and (**c**) 800°C. (d, e, f) TEM images of a nanocrystallite from (a), (b), and (c), respectively.

Typical field emission scanning electron microscopy (FESEM) and transmission electron microscopy (TEM) images of KNbO_3 _powders obtained from heat-treating gels at 600°C, 700°C, and 800°C are shown in Figure 1. Particle sizes were found to be much smaller than those produced by conventional mixed-oxide processing. The 600°C sample in Figure 1a showed that most primary particles were < 50 nm in size, but many of these had clustered into agglomerates. Raising the temperature to 700°C resulted in particle sizes increasing to approximately 70 nm, as shown in Figure 1b. Particles of up to approximately 80 nm in size were present in the 800°C sample shown in Figure 1c. Figure 1d, e, f shows TEM images of nanoscale KNbO_3 _particles in a brick-like morphology. Increasing heat-treatment temperature led to an increase in particle size, which was accompanied by an incremental increase in the brick-like morphology. The average grain size of aggregated KNbO_3 _powders was estimated to be < 100 nm. Table 1 shows average particle sizes obtained at different temperatures estimated from FESEM and TEM images, and the given error was ± 1 standard deviation.

**Table 1:** Particle size dependence on gel heat-treatment temperature

Heat-treatment temperature (°C)	600	700	800
Particle size (nm)	40 ± 10	70 ± 15	80 ± 15

**Figure 2:**
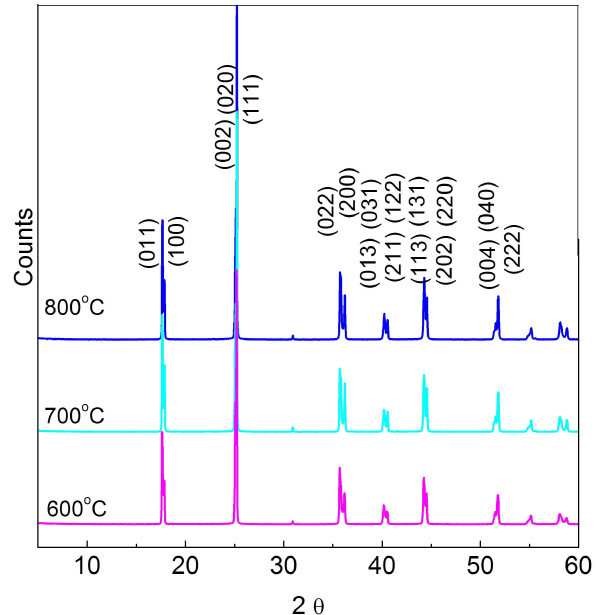
**Synchrotron XRD patterns of nanoscale KNO_3 _powders obtained by heat-treating gels at the stated temperature**. *λ *= 1.2348 Å.

Rietveld refinement results of synchrotron X-ray diffraction (XRD) data for KNO_3 _powders obtained by heat-treating gels at 600°C, 700°C, and 800°C are given in Table 2, and the corresponding XRD patterns are shown in Figure 2. Each powder crystallized in a perovskite phase with an orthorhombic structure (space group Amm2) at room temperature. Orthorhombic KNbO_3 _is thermodynamically stable at room temperature, and orthorhombic KNbO_3 _crystals have potential in applications as ferroelectric and nonlinear optical materials. The ferroelectric orthorhombic phase was retained even for particles smaller than 50 nm.

**Table 2:** Rietveld refinement results of synchrotron XRD data collected at *λ *= 1.2348 Å

Heat-treatment temperature (°C)	600	700	800
Crystal structure	Orthorhombic	Orthorhombic	Orthorhombic
Space group	Amm2	Amm2	Amm2
Unit cell dimensions			
a (Å)	4.004135	4.006313	4.007833
b (Å)	5.737700	5.726862	5.724034
c (Å)	5.742700	5.736795	5.734393
Atomic coordinates	*X*	*Y*	*Z*
O1(4d)	0	0.254	0.285
O2(2b)	0.500	0	0.021
K (2b)	0.500	0	0.517
Nb (2a)	0	0	0

*R*_wp _of all samples was < 10%.

**Figure 3:**
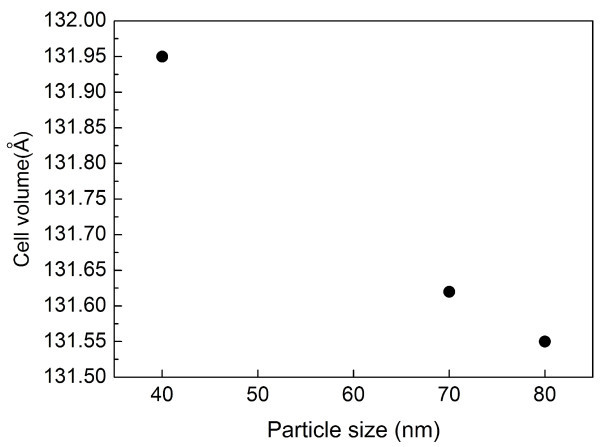
**Rietveld refinement of synchrotron data**. For nanoscale KNO_3 _powders showing cell (**a**) parameters and (**b**) volume.

A cell volume plot is shown in Figure 3, and cell volume increased with decreasing particle size. An increase in unit cell volume has been reported for many metal oxides and ferroelectric materials [19-22]. The most consistent explanation for this in small oxide particles is the effect of the truncated attractive Madelung potential that holds the oxide lattice together [23]. The Rietveld analysis showed increasing distortions with decreasing particle size.

**Figure 4:**
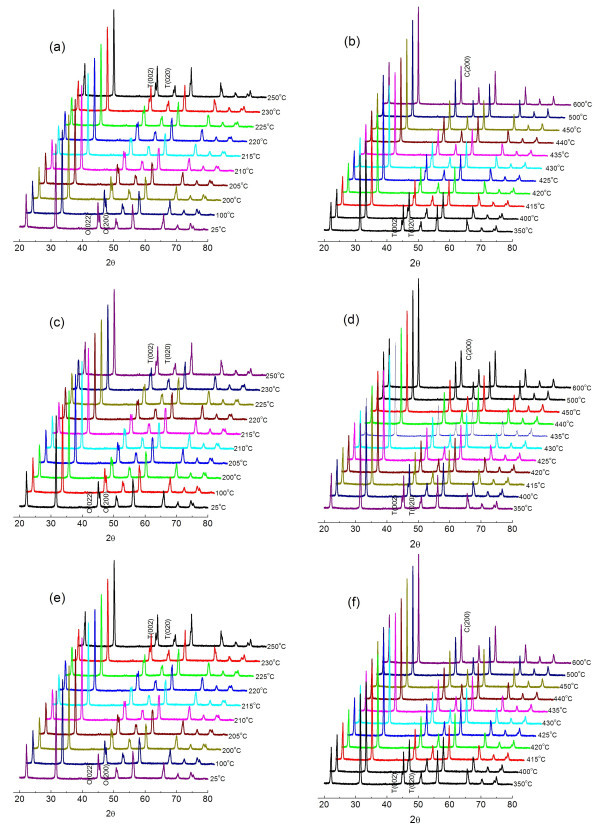
**XRD patterns showing phase transition of nanoscale KNO_3 _powders**. Obtained upon heat-treating gels at different temperatures: (**a**) orthorhombic to tetragonal (600°C), (**b**) tetragonal to cubic (600°C), (**c**) orthorhombic to tetragonal (700°C), (**d**) tetragonal to cubic (700°C), (**e**) orthorhombic to tetragonal (800°C), and (**f**) tetragonal to cubic (800°C).

Figure 4 shows temperature-dependent XRD patterns of nanoscale KNO_3 _powders obtained by heat-treating gels at different temperatures. Three structural types were distinguished by the diffraction at 44° to 46° 2 *θ*. The clearly split peaks were indexed to the 022 and 200 planes for the orthorhombic phase. Broad diffractions of reversed intensity to those of orthorhombic diffractions were considered to correspond to 002 and 020 tetragonal diffractions, since a reversed intensity was observed for the corresponding peaks of the high-temperature tetragonal phase above approximately 220°C. The single peak of the 200 plane was identified as that of the cubic phase above approximately 430°C.

Figure 4 shows that there was no obvious difference in transition temperature between the three samples. Temperature-dependent XRD showed that the actual transition temperature was nearly unchanged, and that the Curie temperature (*T*_C_) and phase transition were independent of particle size.

**Figure 5:**
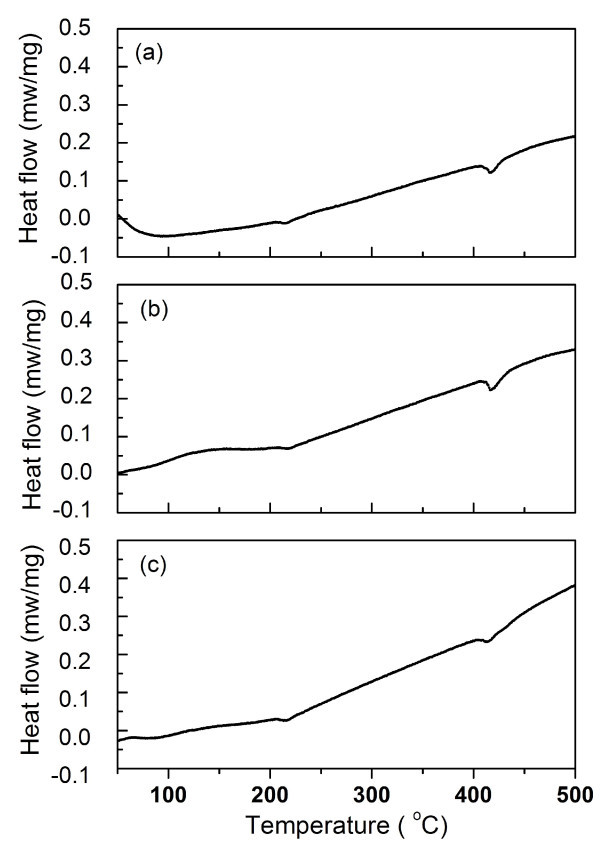
**DSC plots of nanoscale KNO_3 _powders obtained by heat-treating gels**. At (**a**) 600°C, (**b**) 700°C, and (**c**) 800°C.

To further investigate the phase transition of nanoscale KNbO_3_, the differential scanning calorimetry (DSC) analyses were undertaken and the results are shown in Figure 5. Table 3 shows transition temperatures observed from DSC, for the three nanoscale KNbO_3 _samples of different particle sizes. The lower temperature corresponded to the phase change from orthorhombic to tetragonal, and the higher temperature was that from tetragonal to cubic. DSC results showed that phase transition was independent of particle size.

**Table 3:** Transition temperature observed from DSC

Heat-treatment temperature (°C)	600	700	800
Transition temperature (°C)	213.2, 414.5	215.5, 415.7	211.2, 412.5

## Conclusions

Nanoscale KNbO_3 _powders were synthesized using the sol-gel method. The average KNbO_3 _grain size was estimated to be within 100 nm from FESEM and TEM images, and TEM images showed that nanoscale KNbO_3 _particles had a brick-like morphology.

Synchrotron XRD and Rietveld refinement showed that the ferroelectric orthorhombic phase was retained at room temperature, even for particles smaller than 50 nm. Temperature-dependent XRD confirmed that the actual transition temperature was nearly unchanged and that the *T*_C _and phase transition were independent of particle size. Rietveld analysis showed increasing distortions with decreasing particle size.

## Methods

Precursor solutions were prepared using the sol-gel method reported in the literature [24]. K-ethoxide, Nb-pentaethoxide, 2-methoxyethanol, K-ethoxide, and Nb-pentaethoxide were dissolved in 2-methoxyethanol and refluxed at 120°C for 90 min in dry N_2_. The concentrations of all precursor solutions were 0.32 mol/L. Weighed gel samples in Pt cells were calcined at 600°C to 800°C for 3 min in air to obtain crystalline powders, with a heating rate of 10°C/min.

Powder sizes and morphologies were examined using FESEM (JEOL JSM-7500F; JEOL Ltd., Tokyo, Japan) and TEM (JEOL JEM-2010; JEOL Ltd.). Crystal structures were determined using high-resolution synchrotron radiation diffractometry at the BL14B1 beam line of Shanghai Synchrotron Radiation Facility, using 1.2398 Å X-rays with a Huber 5021 6-axes diffractometer (energy = 3.5 GeV). Structural refinements were performed using the Rietveld analysis program X'Pert Highscore Plus (PANalytical X-ray Company, Almelo, The Netherlands). Phase transitions were investigated using non-ambient XRD (PANalytical X'pert Pro, Cu Kα, 40 kV, 40 mA) with a Pt strip stage from ambient temperature to 600°C. The differential scanning calorimetry (NETZSCH STA 449F3, Selb, Germany) was used to follow the phase transitions. Nitrogen was used in the DSC measurement at a flow rate of 50 ml/min with a heating rate of 5°C/min. The measurement was carried out in the temperature range of 50°C to 500°C.

## Abbreviations

DSC: differential scanning calorimetry; FESEM: field emission scanning electron microscopy; KNbO_3_: potassium niobate; TEM: transmission electron microscopy; XRD: X-ray diffraction.

## Competing interests

The authors declare that they have no competing interests.

## Authors' contributions

HC performed the sample preparation, analyzed the materials, and interpreted the results. YZ participated in the XRD, FESEM, TEM, and DSC measurements. YL participated in the synchrotron XRD measurements. All authors read and approved the final manuscript.
